# Supplementary material for: Drug repurposing for aging research using model organisms
Source: Aging Cell. 2017 Jun 16;16(5):1006–15. doi: 10.1111/acel.12626 (PMC5595691; doi:10.1111/acel.12626)
Supplement: Supplementary file 7 — Data S1 Zip‐Archive of all report cards. [file ACEL-16-1006-s007.zip › RC_DAO.pdf]

DAO

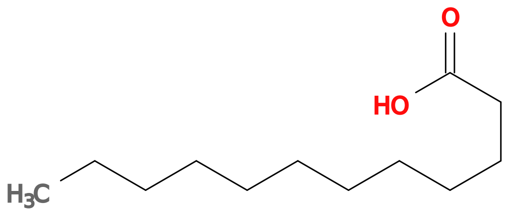

Database identifiers

ChEMBLCompound	CHEMBL108766
CHEBI	30805
eMolecules	495322

Ranking

	Rank	Score
Drosophila	333/697	0.461
C. elegans	196/591	0.249

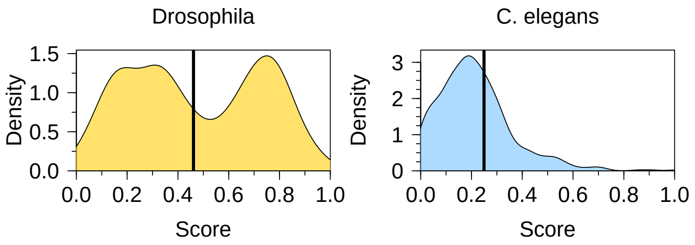

	Ageing implication	Domain conservation	Binding site conservation	Binding affinity	Bioavailability	Lipinski	Promiscuity	Purchasability	Drug approval	**Total**
Drosophila	0.792	0.96	1.0	0.419	(0.9)	0.0	-0.0	0.1	0.075	0.461
C. elegans	0.792	0.953	0.973	0.419	0.243	0.0	-0.0	0.1	0.075	0.249

Names

- **dodecanoic acid**
- 1-undecanecarboxylic acid
- ABL
- C12 fatty acid
- C12:0
- Ch3-ch210-cooh
- Coconut oil fatty acids
- dodecoic acid
- Dodecylcarboxylate
- Duodecylic acid
- Lauric acid
- Laurinsaeure
- Laurostearic acid
- Vulvic acid

Roles

plant metabolite, antibacterial agent, algal metabolite

Status

Approved drug (according to ChEMBL)	No
Number of Rule of 5 violations	0
Binding affinity to original target in log units (RF-Score prediction)	4.67
Burns *C. elegans* bioavailability prediction	-2.43

Compound Target Characteristics

Alcohol dehydrogenase class-3

Best gene implication in ageing for this target family came from gene P12711 annotated in UniProt release 2014_02. Annotation GO 7568 (aging) was Inferred from Expression Pattern

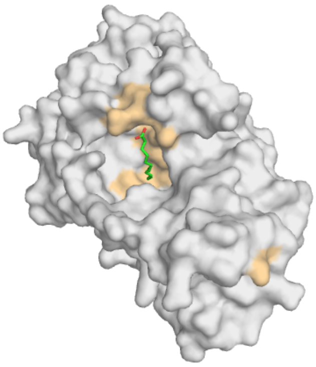

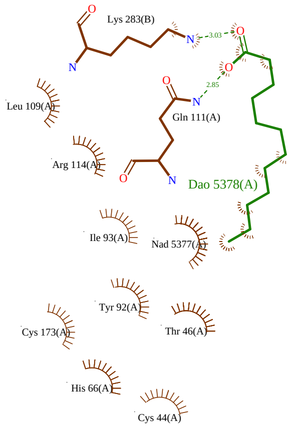

protein	amino acids contacts (binding site)
PDB:1ma0:chainA:P11766	C T H Y I L Q R C K
tr:Q6IRT1:Q6IRT1_HUMAN	C T H Y I L Q R C K
sp:P11766:ADHX_HUMAN	C T H Y I L Q R C K
sp:P12711:ADHX_RAT	C T H Y I L Q R C K
sp:P28474:ADHX_MOUSE	C T H Y I L Q R C K
tr:Q6P5I3:Q6P5I3_MOUSE	C T H Y I L Q R C K
sp:P46415:ADHX_DROME	C T H Y I L Q R C K
sp:Q17335:ADHX_CAEEL	C T H Y V L Q R C K
sp:P32771:FADH_YEAST	C T H Y T L G R C K

	whole protein		domain-based		contact-based	
protein	ident	simil	ident	simil	ident	simil
PDB:1ma0:chainA:P11766	1.0	1.0	1.0	1.0	1.0	1.0
tr:Q6IRT1:Q6IRT1_HUMAN	1.0	1.0	1.0	1.0	1.0	1.0
sp:P11766:ADHX_HUMAN	1.0	1.0	1.0	1.0	1.0	1.0
sp:P12711:ADHX_RAT	0.94	0.98	0.96	0.99	1.0	1.0
sp:P28474:ADHX_MOUSE	0.93	0.98	0.94	0.98	1.0	1.0
tr:Q6P5I3:Q6P5I3_MOUSE	0.93	0.98	0.94	0.98	1.0	1.0
sp:P46415:ADHX_DROME	0.7	0.9	0.74	0.92	1.0	1.0
sp:Q17335:ADHX_CAEEL	0.67	0.88	0.73	0.9	0.9	0.97
sp:P32771:FADH_YEAST	0.6	0.84	0.65	0.87	0.8	0.84

Fdh (FBgn0011768) associated phenotypes

heat sensitive, memory defective

(Information from FlyBase)

Fdh (UniProt:P46415) annotation

Function: Class-III ADH is remarkably ineffective in oxidizing ethanol, but it readily catalyzes the oxidation of long-chain primary alcohols and the oxidation of S-(hydroxymethyl) glutathione.

Cofactor:Zn(2+)Note=Binds 2 Zn(2+) ions per subunit. ;

(Information from UniProt)

H24K24.3 (UniProt:Q17335) annotation
Function: Class-III ADH is remarkably ineffective in oxidizing ethanol, but it readily catalyzes the oxidation of long-chain primary alcohols and the oxidation of S-(hydroxymethyl) glutathione.
Cofactor: Zn(2+)Note=Binds 2 Zn(2+) ions per subunit. ;
Subunit: Homodimer.
Subcellular location: Cytoplasm

(Information from UniProt)
